# From structure to the dynamic regulation of a molecular switch: A journey over 3 decades

**DOI:** 10.1016/j.jbc.2021.100746

**Published:** 2021-05-03

**Authors:** Susan S. Taylor, Jian Wu, Jessica G.H. Bruystens, Jason C. Del Rio, Tsan-Wen Lu, Alexandr P. Kornev, Lynn F. Ten Eyck

**Affiliations:** 1 Department of Pharmacology, University of California at San Diego, San Diego, California, USA; 2 Department of Chemistry and Biochemistry, University of California at San Diego, San Diego, California, USA; 3 Department of Biochemistry and Biophysics, University of California at San Francisco, San Francisco, California, USA; 4 San Diego Supercomputer Center, University of California at San Diego, San Diego, California, USA

**Keywords:** protein kinases, protein structure, crystallography, cAMP, dynamics, allostery, intrinsically disordered regions, cAMP-dependent protein kinase (PKA), catalytic subunit, regulatory subunit, AKAPA, kinase anchoring protein, CNB, cyclic nucleotide binding, DD, dimerization/docking, LSP, Local Spatial Pattern, PDB, Protein Data Bank, PKA, protein kinase, SAXS, small-angle X-ray scattering

## Abstract

It is difficult to imagine where the signaling community would be today without the Protein Data Bank. This visionary resource, established in the 1970s, has been an essential partner for sharing information between academics and industry for over 3 decades. We describe here the history of our journey with the protein kinases using cAMP-dependent protein kinase as a prototype. We summarize what we have learned since the first structure, published in 1991, why our journey is still ongoing, and why it has been essential to share our structural information. For regulation of kinase activity, we focus on the cAMP-binding protein kinase regulatory subunits. By exploring full-length macromolecular complexes, we discovered not only allostery but also an essential motif originally attributed to crystal packing. Massive genomic data on disease mutations allows us to now revisit crystal packing as a treasure chest of possible protein:protein interfaces where the biological significance and disease relevance can be validated. It provides a new window into exploring dynamic intrinsically disordered regions that previously were deleted, ignored, or attributed to crystal packing. Merging of crystallography with cryo-electron microscopy, cryo-electron tomography, NMR, and millisecond molecular dynamics simulations is opening a new world for the signaling community where those structure coordinates, deposited in the Protein Data Bank, are just a starting point!

The scientific community is enormously grateful to the visionary leaders of the early crystallography community who realized how important it would be to have an international resource where all new structures would be deposited and validated. Max Perutz, Michael Rossmann, and Fred Richards were among the early pioneers who championed the PDB concept. In 1971 the Protein Data Bank (PDB) was officially announced in *Nature New Biology* (1) as a joint venture between Brookhaven National Laboratory under the direction of Walter Hamilton and the Cambridge Crystal Data Center, founded by Olga Kennard, with an initial holding of seven protein structures. Helen Berman, who was also involved with the initial establishment of the PDB, led the transformation of the PDB into a modern, worldwide database in 1998. The determination and dedication of these two pioneering women, along with the visionary leadership of Perutz, Richards, Rossmann, and others, was essential to the founding of the PDB and to the development of the modern PDB. From an initial concept as a static archive to the development of a dynamic research tool, the development of the PDB allows the research community to leverage the investments made by many funding agencies across disciplinary and national boundaries and solidifies the concept that these structures belong to the community and not just to the individual laboratory that solved the structure. It represents an exciting commitment to science and to the scientific community. Of course, no one envisioned the ways in which this community would explode along with the technologies in computing over the ensuing decades. We describe here the impact that this vision has had on the signaling community using cAMP-dependent protein kinase (PKA) as a model. We not only highlight the wide-ranging benefits that have evolved but also emphasize future challenges and new opportunities to build on for the future as our understanding of proteins evolves from those early days of the first crystals of hemoglobin, lysozyme, and lactate dehydrogenase.

## PKA catalytic subunit

The structure of the PKA catalytic (C) subunit was our first entry into the PDB in 1991. Taylor had been trained in protein chemistry and structural biology at the MRC Laboratory of Molecular Biology in Cambridge, and Michael Rossman was one of her major mentors in her early career when she was sequencing lactate dehydrogenase. Lynn Ten Eyck with his long-term commitment to public data bases was also a part of the team as was Janusz Sowadski who led the crystallography. So, there was never a question in their minds not to share this structure with the signaling community that was just beginning to make inroads into the structure of protein kinases, which are now recognized to represent one of the largest gene families and were already associated with many diseases. Ours was the first protein kinase structure to be solved, and even before the structure was published, we were anxious to share this new information with our colleagues such as Bruce Kemp and others. A core philosophy of my laboratory has been that one needs a structure before one can truly begin to understand function, and so for me having a structure was a starting point.

**Figure 1:**
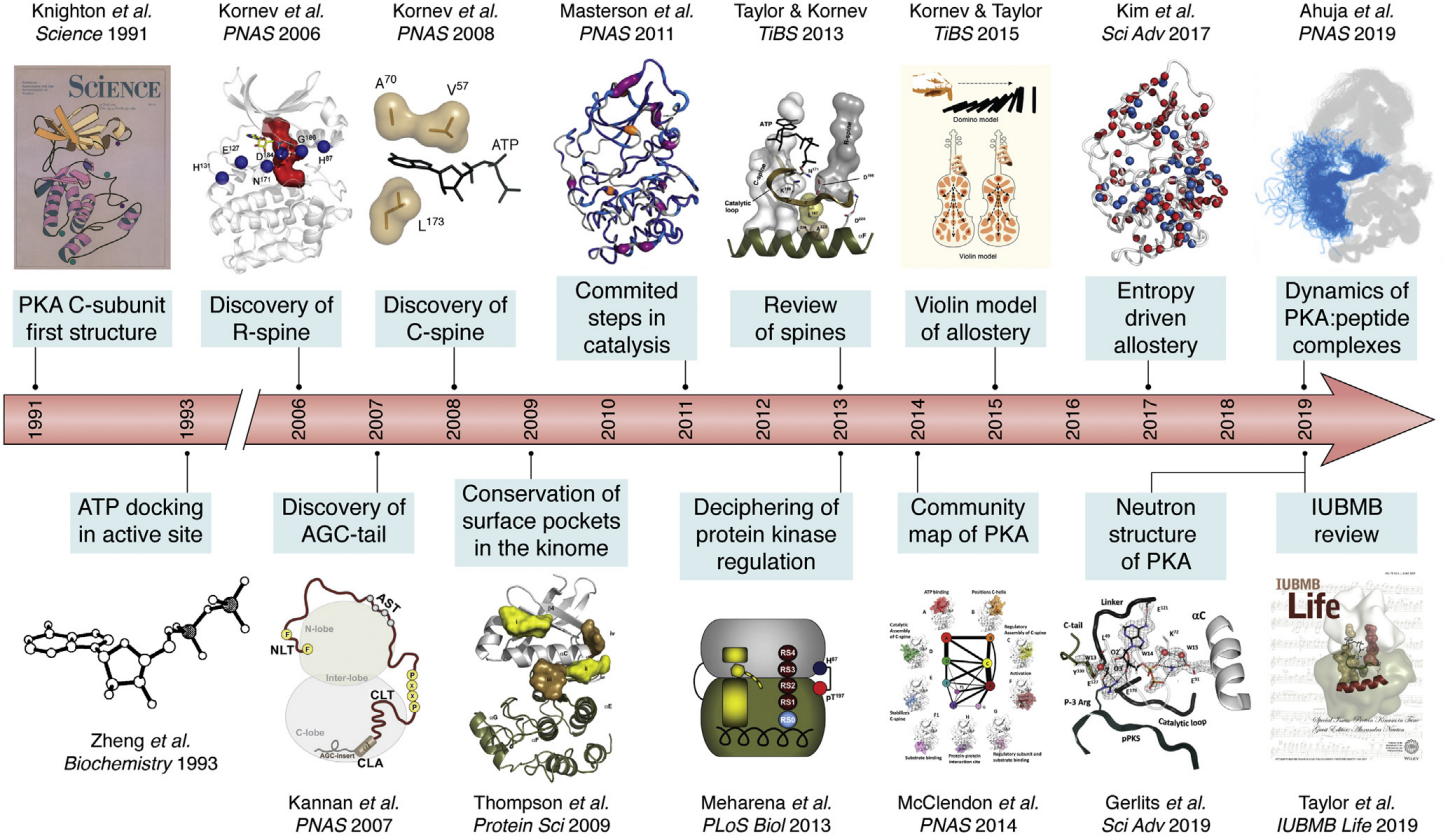
**Evolution of the kinase domain (1991–2021).** The first structure of the PKA C-subunit in 1991 defined the fold of a fully active kinase and the docking of a high-affinity pseudosubstrate peptide derived from the heat-stable protein kinase inhibitor (2, 3). The next structure in 1993 defined the intricate way in which ATP was docked into the active site cleft (4). The full appreciation of the dynamics of the C-subunit and its evolution as a dynamic molecular switch unfolded over the next 3 decades. Discovery of R-Spine, 2006 (9); Discovery of AGC-tail as a conserved feature of the AGC family, 2007 (22); Discovery of the C-spine, 2008 (10); Identification of conserved surface pockets in the kinome, 2009 (51); Elucidation of committed steps in catalysis by NMR, 2011 (11); TiBS review of spines, 2013 (52); Deciphering protein kinase regulation, 2013 (53); Community maps, 2014 (14); Violin model of allostery, 2015 (54); Entropy-driven Allostery revealed by NMR, 2017 (12); Zooming in on protons with Neutron Diffraction, 2019 (55); Dynamics of PKA:peptide complexes, 2019 (56); IUBMB review, 2019 (57).

**Figure 2:**
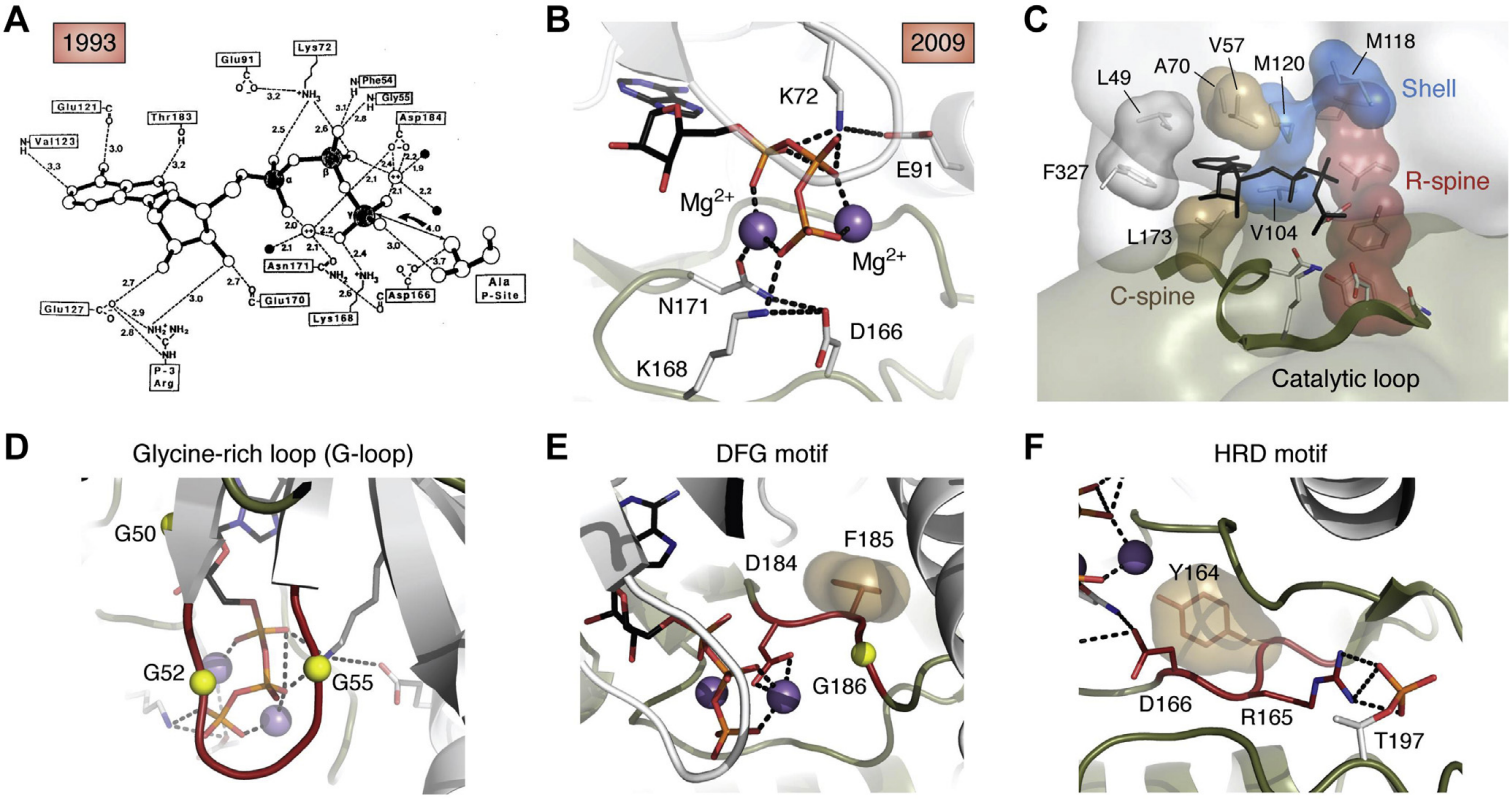
**ATP binding as a driver of kinase dynamics.** The PKA C-subunit not only defined a novel fold but also defined a novel ATP-binding site that was distinct from the Rossmann fold. The importance of the hydrophobic shell that embraces the nucleotide unfolded over many decades. *A*, the AT- binding site defined in 1993 (4) showed the intricate network of hydrogen bonding and electrostatic interactions that bury the entire nucleotide in a closed conformation. *B*, the second metal ion is essential for the synergistic high-affinity binding of ATP in the protein kinase inhibitor complex and for phosphotransfer (7, 8, 13). *C*, expanded hydrophobic shell that surrounds the ATP includes the C-spine (*tan*) (10), the R-spine (*red*) (9), and the shell (*blue*) (53) as well as additional residues from the N-lobe and C-tail (58). This shell defines the entropy-driven allostery that drives the phosphor transfer mechanism (12, 52, 54). *D*, conserved motifs of the kinase core. *Left*, glycine-rich loop (G-Loop) is a conserved feature of the protein kinases. *Middle*, DFG motif. D binds to the second metal ion in the ATP complex, and F is part of the R-Spine (RS3). G provides flexibility and is part of β strand 9 that is then followed by the activation loop. *Right*, HRD Motif. This motif contains an R-spine residue (RS1) and defines a key allosteric node. In some kinases such as PKA the H is conserved as a Tyr. The H/Y residue reaches across and stabilizes the catalytic loop, the R reaches out to the activation loop phosphorylation site (pT197 in PKA), and the D is the catalytic base that is positioned at the site of phosphor transfer.

Although we did not fully appreciate it at the time, we were very fortunate with that first structure to have captured an active and fully phosphorylated protein kinase (Fig. 1). The bilobal fold was novel as was the ATP-binding pocket (2). Although ATP was present in the buffer of that initial structure and the position of the pseudosubstrate peptide derived from the heat-stable protein kinase inhibitor was clearly defined (3), the position of the nucleotide was only predicted to be localized to the cleft between the two lobes. In the subsequent structure when we used a 10-fold excess of Mg^2+^ over ATP we were able to trap both the ATP along with two Mg^2+^ ions and the peptide (Fig. 2*A*) (4). This represented a novel ATP-binding site that is defined by the glycine-rich loop (G-Loop) (Fig. 2*D*) that embraces the adenine ring of ATP and helps to secure it at the base of the cleft as well as to position the γ-phosphate for transfer to a protein substrate. It is quite distinct from the P-Loop (5) or the Walker motif that had been described by Rossmann for binding nucleotides including ATP in hexokinase and NAD in the dehydrogenases (6). So, we captured an enormous amount of information in those first structures; however, we also completely missed or failed to appreciate some of the most essential features of the C-subunit. Although both metal ions were captured in that 1993 structure, we did not appreciate the crucial importance of the second metal ion as the “lynchpin” that is essential for high-affinity binding of ATP and for the release of the nucleotide (Fig. 2*B*) (7, 8). Most other protein kinase structures still do not properly capture or grasp the importance of that second metal ion. What was also missing from this original structure was an appreciation of the flexibility and malleability of this protein kinase structure and the appreciation of the concept that protein kinases had evolved not to be efficient catalysts but to be dynamic molecular switches.

## Defining the protein kinase molecular switch (1991–2021)

In contrast to metabolic kinases such as hexokinase, which have evolved to be efficient catalysts, the protein kinases have evolved to be molecular switches. They are relatively poor catalysts. To understand and fully appreciate the switch mechanism that drives the assembly of an active kinase required many protein kinase structures as well as an interdisciplinary approach that went beyond the static structures that are captured in each crystal lattice (Fig. 1). Over the following decade after our structure of that first protein kinase was solved there were many protein kinases, all deposited in the PDB, and almost all were solved by molecular replacement. Some were active conformations, whereas most were inactive conformations. As a way to potentially discover some of the fundamental features required for activation, we used a computational tool, Local Spatial Pattern (LSP) alignment (9). This spatial alignment where we compared active and inactive kinases, led to the discovery of a fluid “Regulatory Spine” that appeared to be the hallmark signature of every active kinase. The four R-spine residues were part of several of the well-recognized conserved motifs such as the HRD motif (Fig. 2*E*) that precedes the catalytic loop and the DFG motif (Fig. 2*F*) that follows the Mg Positioning Loop in the C-Lobe as well as the C-Helix and β strand 4 from the N-Lobe. The essential feature of the R-spine is that it is dynamically assembled, often in response to phosphorylation of the activation loop. Other conserved motifs were embedded in the Catalytic Spine (C-Spine) that is aligned in a parallel manner to the R-spine and is completed by the adenine ring of ATP (10). The importance of this hydrophobic core for entropy-driven allostery was only fully appreciated experimentally when Veglia was able use NMR to capture the side chain dynamics of these hydrophobic residues (11, 12).

The trajectory that defines our progress and what we learned over the intervening 3 decades since that initial structure is summarized in Figure 1, whereas our enhanced understanding of the active site cleft and ATP binding is summarized in Figure 2, which also illustrates some of the key conserved motifs that are shared by all protein kinases. From the first structures we defined all of the hydrogen-bonding interactions (Fig. 2*A*) and the conserved motifs (Fig. 2, *D*–*F*), but we completely failed to appreciate the hydrophobic shell that embraces the active site with the adenine ring captured at the base of the cleft between the two lobes and the γ-phosphate trapped in a fully closed conformation poised for transfer to a protein substrate at the edge of the cleft (Fig. 2*C*). This organization of the nucleotide with the G-Loop (Fig. 2*D*) is completely different from the P-Loop structures that include both the GTPases and the ATPases, where it is the γ-phosphate that is trapped at the base of the cleft. Also captured in our first structures were the two metal-binding sites (Fig. 2*B*), although we failed completely to appreciate the importance of the second “lynchpin” metal ion (7) as being essential for catalysis and for capturing the high-affinity binding of ATP (8, 13). Our subsequent “Community Map” analysis of the C-subunit goes beyond those conserved features of the secondary structural elements and shows how the kinase domain is organized in discreet “communities” that are committed to specific functions (14). The correlated motions of the hydrophobic side chains that commit the kinase to transfer of the phosphate are experimentally captured by NMR and in the simulations. With these tools we are able to create a dynamic portrait of that static structure that was captured in the crystal.

## Isoform diversity

**Figure 3:**
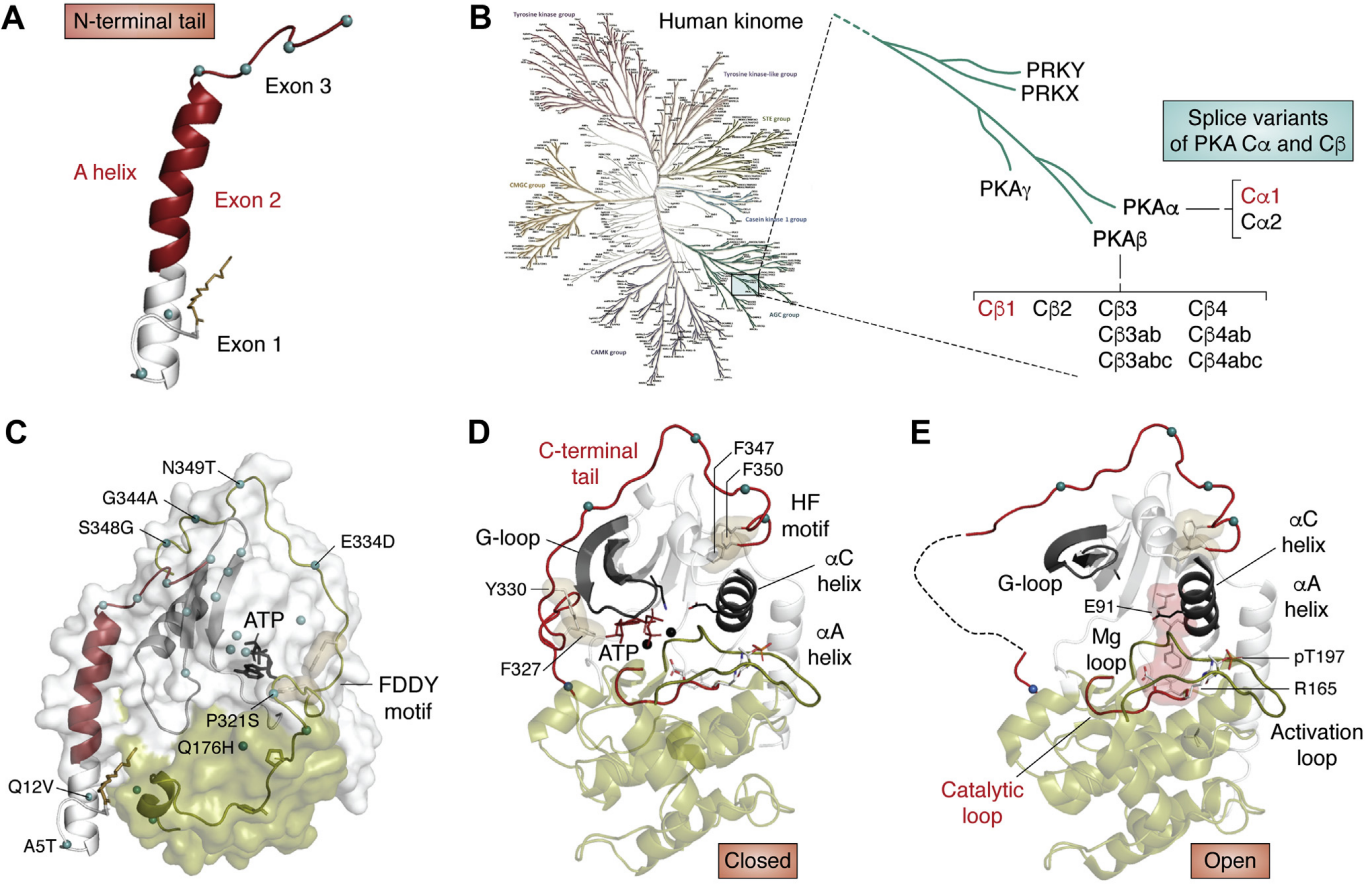
**Isoform diversity of the catalytic subunit.** The functional and spatial nonredundancy of the Cα and Cβ isoforms expands the size of the PKA subfamily. Our growing recognition that each splice variant is localized differently and committed to a specific function has the potential to greatly expand the size of the kinome. *A*, the N-terminal tail of the C-subunit is encoded by Exons 1, 2, and 3. *B*, splice variants of the PKA Cα and Cβ subunits (20). Several oncogenic fusion proteins have been identified where exon 1 of Cα and/or Cβ is replaced by another domain such as the J Domain of DNAJB1 (59) or the N terminus of the ATPase 1 (60). *C*, N-terminal and C-terminal tails wrapped around both lobes of the conserved kinase core. *D*, the C-terminal tail also contains many of the residues that differs between Cα1 and Cβ1 (indicated as *blue dots*). Like the N-terminal tail, the C-tail reaches around both lobes of the kinase core. In the ATP-bound closed conformation the C-tail is firmly anchored to the core through hydrophobic interactions of F^327^ and Y^330^ with the adenine ring of ATP and through the hydrophobic motif (F^347^ and F^350^) that anchors the C terminus to the αC-Helix. *E*, in the open apo conformation a portion of the C-Tail becomes disordered and the G-Loop assumes an open conformation.

Increasingly we are appreciating also that each kinase often consists of separate isoforms as well as splice variants. Typically, these variants are functionally nonredundant, so indeed Hunter’s prediction of 1001 kinases is most likely a vast underestimate (15). In the case of PKA there are three isoforms on the kinome tree (16) (Fig. 3). Almost everything that we know about PKA, which in so many ways has served as a poster child for the protein kinase superfamily, is about the Cα1 subunit (PRKACA1), which was cloned in 1986 (17). The Cγ subunit (PRKACG) is highly specialized and localized to testes and sperm (18). In contrast, the Cβ1 subunit (PRKACB1) cloned in 1986 (19), is found in most tissues, although in many cases at a lower level than Cα1. However, the Cβ gene also has many splice variants at the first exon (20, 21) (Fig. 3*B*), and these are expressed in a highly tissue-specific manner. Many of the sequence differences between Cα1 and Cβ1 are localized to the N-terminal tail and the C-terminal tail (Fig. 3*C*) that both embrace and regulate the conserved kinase core that is shared by all protein kinases. As with the PKA regulatory subunits where there are four separate genes that code for four functionally nonredundant proteins, it is likely that the splice variants of Cβ are also functionally nonredundant. Nearly half of PKA expressed in the brain, for example, is accounted for by Cβ isoforms. Some such as Cβ3 and Cβ4 are expressed exclusively in brain, whereas Cβ2 is predominant in lymphoid tissues. The combinatorial diversity that is introduced by these Cβ splice variants suggests that these proteins should be considered as separate proteins, which adds challenging complexity to the kinome.

## PKA holoenzymes trap the active kinase in an inhibited state

One of the most important questions facing the kinase signaling community is how to capture the physiologically relevant inhibited state of any kinase. In the case of PKA we are fortunate in that the kinase is inhibited by cAMP-binding regulatory (R) subunits that are encoded by separate genes. Almost all other protein kinases are locked into an inhibited state by domains that are fused to the kinase domain, typically to the N terminus of that domain, making them experimentally challenging for the crystallographer. For this reason, most of the kinases in the PDB represent isolated kinase domains that are missing so many of the key regulatory features that are embedded in the flexible linkers and domains that flank the kinase domains. Cryo-electron microscopy is opening a new opportunity for the signaling community in that it is allowing us to look at these larger full-length multidomain proteins.

If we compare the tyrosine and tyrosine-like protein kinases with the AGC kinases there is a fundamental difference. The protein tyrosine kinases in the absence of activation appear to be mostly locked into an inhibited state by their flanking domains, and assembly of the R-spine only occurs after activation. In spite of their fundamental importance of the nonkinase domains for activation, in large part because of the complex organization and flexibility of the nonkinase domains, it has been especially challenging to trap this inhibited state. All of this essential information is missing when one looks only at the kinase domain. Instead, one sees the activation loop mostly in a disordered state. In contrast, the AGC kinases are first assembled as an active kinase typically by ordering the C-terminal tail through multiple phosphorylation events that can involve hetero kinases as well as autophosphorylation (22). The active kinase is then trapped in an inactive state that can be “unleashed” by second messengers such as calcium, cAMP, cGMP, diacyl glycerol, and IP3 lipids.

For PKA there are four functionally nonredundant R-subunits (RIα, RIβ, RIIα, RIIβ), and each holoenzyme seems to capture the C-subunit in a different way creating a variety of allosteric mechanisms that all lead to allosteric activation by cAMP (23). The R-subunits are maintained as a dimer by an N-terminal dimerization/docking (DD) domain, which leaves the two cyclic nucleotide binding (CNB) domains in each protomer in close proximity. In addition to trapping the R-dimer, the DD domain serves as a scaffold for A kinase anchoring proteins (AKAPs), which each contain an amphipathic helix that docks the kinase to specific sites within the cell (24). In this way one creates specific “cAMP signaling islands,” each committed to a specific function. Although we have captured R:C complexes that lack the DD domain, it has been challenging to see the localization of the DD domains even when they are present.

To illustrate how our understanding of the PKA R-subunits evolved we use RIα as an example beginning with the structure of an RIα monomer that defined the cyclic nucleotide-binding sites (25). The subsequent structure of the full-length RIα dimer (26) made us appreciate the profound importance of a novel motif that we had originally attributed to crystal packing while disease mutations in RIα are further extending our appreciation of this motif and its implications for PKA signaling.

## cAMP-mediated regulation of PKA signaling leads to the discovery of a new protein interface motif

**Figure 4:**
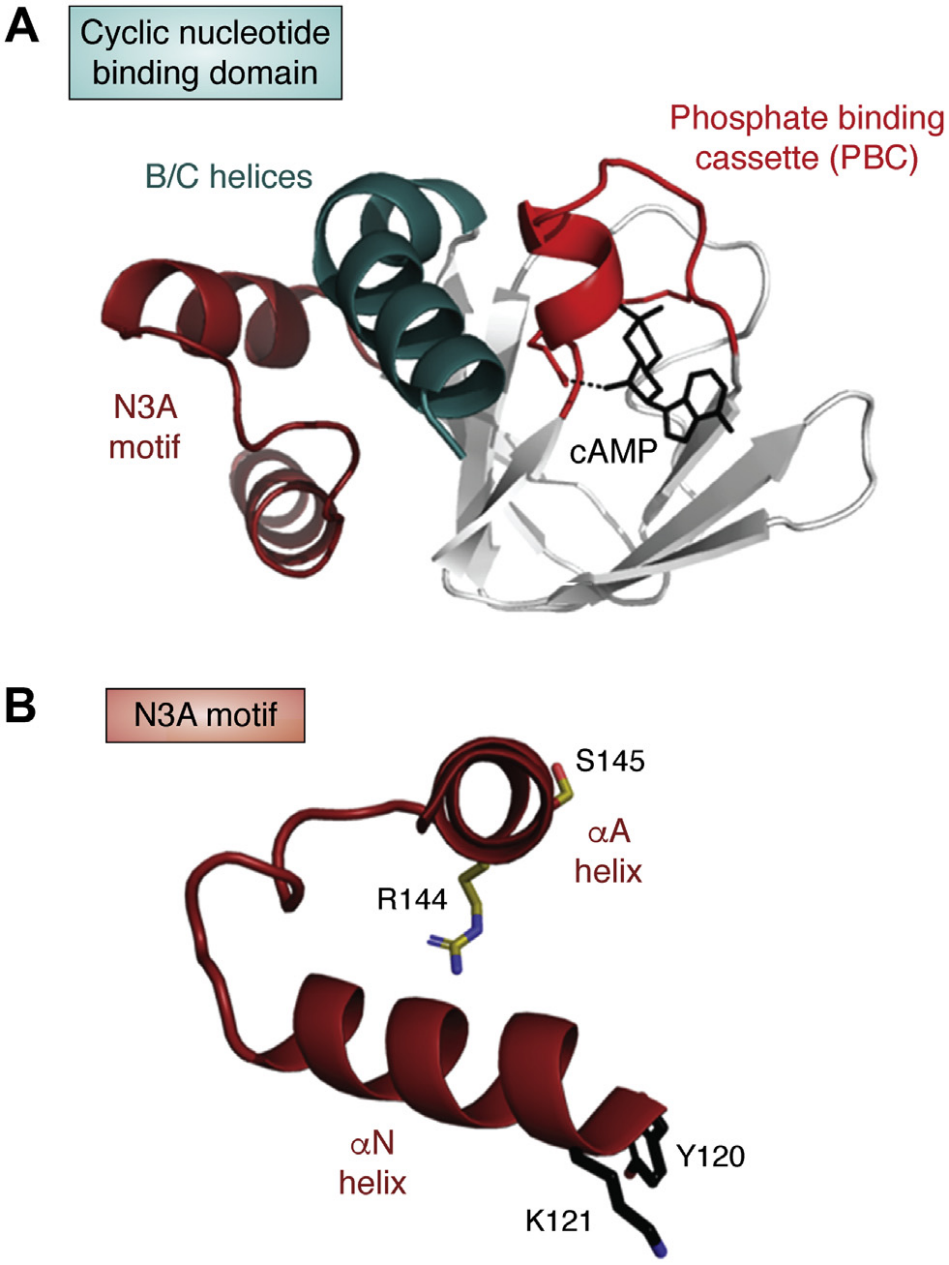
**Evolution of the cyclic nucleotide binding (CNB) domain: Although the cAMP-binding sites were captured in the crystal structure of a deletion mutant of the RIα subunit** (25)**, the full domain architecture and in particular the N3A motif was not recognized or appreciated as a functional part of the eukaryotic CNB domain until the LSP analysis was done** (9)**.***A*, the dynamic features of the CNB domain and in particular the correlated motions of the helical domains are highlighted and further defined in the movie (see Supporting information). The N3A motif that includes the αA and αN helices are in *dark red*, the B/C helix is *teal*, and the phosphate-binding motif that is the signature motif of this domain and binds to the phosphate of cAMP is in *red*. *B*, the key features of the N3A motif are summarized. Two CNC mutations R144S and S145G/D are also indicated.

The most ubiquitous of the four R-subunits is RIα, and it has also been the most studied by a variety of techniques that include crystallography (25), NMR (27), and single molecule optical tweezers (28). It is also the subunit that is most highly mutated in diseases such as Carney Complex (CNC) and *Acrodysostosis* (ACRDYS) (29, 30). In our initial structure of a deletion mutant of the RIα subunit we captured both CNB domains (25) and later compared them with the catabolite activator protein in *E. coli*, a well-known modulator of cAMP-mediated gene transcription in prokaryotes (31). Each CNB domain includes an A Helix that is fused to an eight-stranded β sandwich motif that is followed by two additional helices, αB and αC. The phosphate-binding cassette, which contains the signature recognition motif for cAMP is embedded in a loop between β strands 5 and 6 and also includes a short helix that is an interaction site for the C-subunit. The subsequent structures of two R:C complexes allowed us to appreciate the conformational changes that result when the cAMP-free R-subunit is wrapped around the C-subunit trapping it in an inactive state (32, 33). We subsequently carried out a more comprehensive structure-based analysis that included our LSP method to identify spatially conserved motifs. This allowed us to define the correlated motions of the helical domains that are associated with cAMP binding that lead to the activation of PKA (Fig. 4*A*) (34). In addition to providing a mechanistic understanding of the correlated motions of the helices in the CNB domains, the LSP alignment led to the discovery of an important and completely novel motif that had been overlooked in our initial analysis of the CNB domains and that also distinguishes the eukaryotic R-subunits from catabolite activator protein (34). This motif, referred to as the N3A motif (Fig. 4*B*), includes an additional helix at the N terminus and constitutes a classic helix–turn–helix motif. A 3^10^ helix is embedded in the linker that joins the two helices. There are two N3A motifs in each R-subunit. One is fused to the linker that joins the CNB-A to the N-terminal dimerization domain, and the other fuses the CNB-A domain directly to the CNB-B domain. Although two CNC mutations are embedded in the first N3A motif, we did not fully appreciate the significance of the N3A motif until we solved the structure of the full-length RIα subunit (26). Originally, we assumed that the dimer interface created by two N3A motifs was simply an artifact of crystal packing. Only with the full-length RIα in hand did we appreciate that the N3A motif was a real and important dimer interface between the two protomers. The significance of this motif has been further amplified by new structures of the full-length RIα holoenzyme and by disease mutations that are embedded within this motif as well as distal CNC mutations that indirectly influence the role of the N3A motif in promoting protein:protein interactions.

### Discovering the N3A motif as a dimer interface

**Figure 5:**
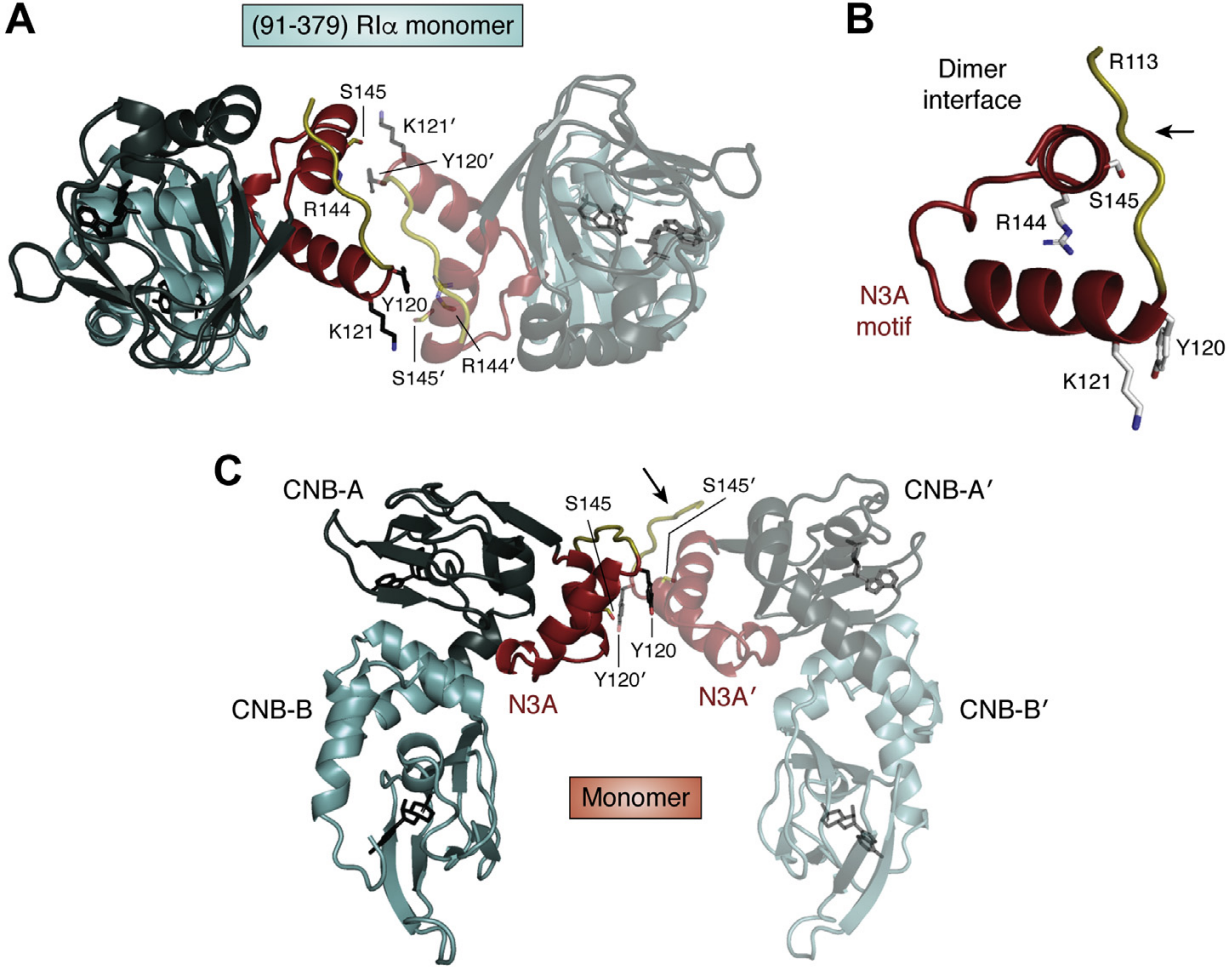
**Crystal packing in the RIα (91–379) structure.** When the first structure of a monomeric RIα was solved the importance of the N3A motif as a defining feature of eukaryotic CNB domains was not recognized. In this structure of RIα (91–379) the N-terminal linker region (residues 92–112) that includes the inhibitor site is disordered and the packing of the N3A motifs between two protomers (N3A:N3A’) was assumed to be an artifact of crystal packing (25). *A*, crystal packing of the two N3A motifs. *B*, dimer interface between the two N3A motifs would leave the two residues that were shown to be drivers of CNC solvent exposed in the monomer. *C*, the interactions between the CNB domains are localized to CNB-A. The *black arrows* indicate the portion of the linker that precedes the N3A motif that is ordered by crystal packing.

**Figure 6:**
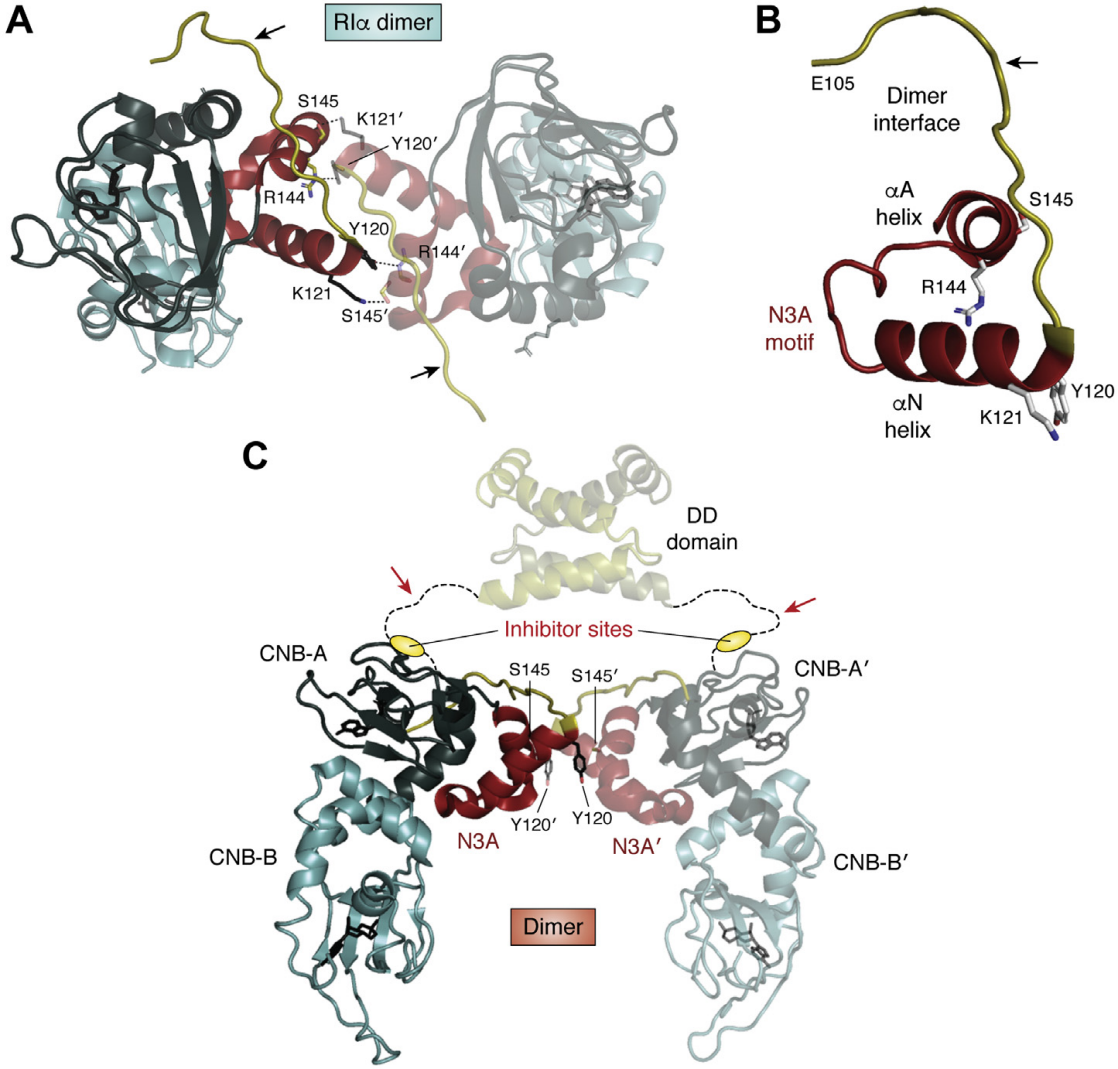
**Structure of the full-length RIα dimer.** The structure of the full-length RIα dimer highlighted the physiological importance of the N3A dimer interface and provided an explanation for the CNC mutations (26). *A*, the packing of the two N3A motifs in each CNB-A domain is a defining feature of the full-length dimer. *B*, the dimer interface is extended in this structure to include residues 108 to 118 and highlights the importance of the segment that precedes the αN helix. *C*, organization of the full-length RIα dimer. Although the DD domain and the linker are present in this protein, these regions are disordered so the cross talk between these residues and the CNB-A domain is not captured. However, the key interaction between the two CNB-A domains explains the compact nature of the RIα dimer, in contrast to the extended conformation of RIIα and RIIβ. It also begins to explain the significance of the CNC mutations. The *black arrows* indicate the extended portion of the linker that precedes the N3A motif that is ordered and part of the extended dimer interface. The *red arrows* indicate intrinsically disordered linker region that includes the inhibitor site (*yellow oval*).

The interface between the two N3A protomers was observed in the initial structure of the RIα (91–379) monomer and was indeed seen in all of our subsequent structures of monomeric RIα subunits; however, we attributed this to crystal packing (Fig. 5). In this structure the six residues preceding the N3A motif become ordered owing to the crystal packing (Fig. 5*B*). It was not until we solved the structure of the full-length RIα dimer (26) (Fig. 6) that we appreciated that this motif was a fundamental feature of the dimer and accounted for the compact structure of the RIα dimer in comparison with the RII dimers, which are fully extended (35). With the RIα dimer structure we began to further appreciate that the dimer interface includes not only the N3A motif itself but also the extended segment that precedes the N3A motif (Fig. 6*C*); this entire region is ordered by interactions between the two protomers. (Fig. 6, *A*–*C*) In the original structure of the RIα monomer only seven additional residues were ordered (113–119) by packing against the opposite protomer (Fig. 5*B*), whereas in the full-length RIα dimer an additional five residues become ordered (Fig. 6*B*). The importance of this segment was appreciated first by NMR (36) and later by optical tweezers (37), which allowed Maillard to explore the thermodynamic properties of the CNB domains. He first captured the thermodynamic properties of the CNB-A domain and later the two CNB domains and then showed how dramatically the properties were altered by the flanking regions. His work emphasized clearly that the N3A motif in the CNB-A domain is a critical driver of the domain dynamics.

The packing of the N3A dimer also begins to explain the importance of the two CNC mutations in this motif (26). In the dimer these two side chains are packed against the opposite protomer, explaining why the Hill coefficient for cAMP activation is reduced from 1.8 to 1.9 to 1.4 to 1.5 with each of these two mutations. Introducing a more severe change at the interface such as Tyr120A or Lys121A actually appears to break the dimer as evidenced by small-angle X-ray analysis and the Hill coefficient is reduced to 1.1 to 1.2.

## Crystal packing

Our analysis of the full-length RIα (Fig. 6) forced us to look more closely at the N3A dimer interface that we had observed in our earlier structures but attributed to crystal packing (25). Indeed, everyone attempts to distinguish biologically important interfaces from crystal packing artifacts. Which is a biologically relevant interface, and which is an artifact of crystal packing? Our experiences with RIα have forced us to think of crystal packing instead as a treasure chest showing us what is possible. The idea that one interface is right and one is wrong is not a correct assumption. Crystal packing tells us what is feasible, and when these interfaces are combined with disease mutations these crystal packing interfaces can tell us which are indeed physiologically relevant. Furthermore, the physiological relevance of these interfaces can be validated by mutagenesis. From Markov State Models we see in the case of RIα that there is an ensemble of structures not a simple two-state model (38). In contrast to the dimerization domain at the N terminus, which is a high-affinity interaction in the RII subunits and actually stabilized in the RIα subunit by two interchain disulfide bonds (39), the interactions between the two CNB domains are lower affinity and do not trap the dimer by size exclusion gel filtration. The physiological relevance is nevertheless captured well by small-angle X-ray scattering (SAXS). In the case of RIα the disulfide bonds in the DD domain are actually thought to be reduced in the resting state (40, 41). The disulfide bonded dimer can be induced by oxidative stress or by AKAP binding, so N3A-mediated cross talk is likely to be more complex in the RI dimers and in the RI holoenzymes.

## Propensity for polymerization

**Figure 7:**
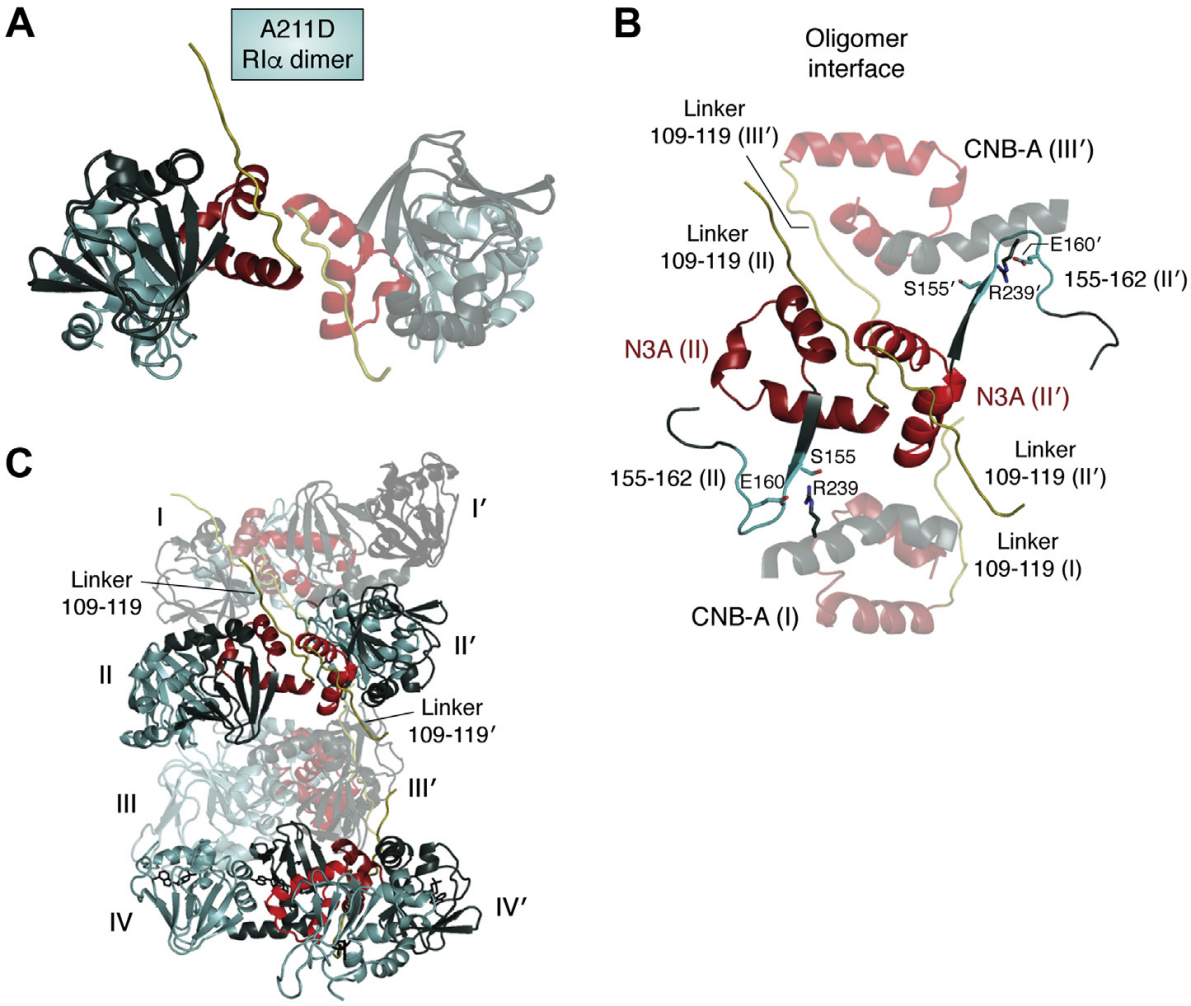
**Propensity for further polymerization is captured in the A211D CNC mutant.***A*, the packing of two N3A motifs in A211D mutant, which is the same feature as the full-length WT dimer. *B*, the potential of the extended N3A motif is captured in this A211D mutant. In this mutant the N-terminal extension that is fused to the N helix in the CNB-A domain mediates one dimer interface as shown in Figure 7; however, the segment that extends from the A helix and includes β strands 1 and 2 of the β subdomain mediates a different dimer interface with another protomer. *C*, the asymmetric unit in this structure includes four dimers and shows how a polymer can form that has features that resemble the Aβ amyloid fibers (42).

Based on NMR data and the structure of a new CNC mutant of RIα an additional polymerization state of RIα has been reported, and this builds further on the N3A (42). The NMR data of this CNC mutant identify “hot spots” that promote polymerization similar to Aβ amyloids, and these target to regions that flank both the N- and C-terminal regions of the N3A motif in CNB-A (Fig. 7). This mutant has a reduced affinity for the C-subunit and is proposed to isolate a complex where the C-subunit is binding in a noncanonical way that keeps it in an active state. A new physiological state was also recently described for the RIα subunit that correlates with LLPS puncta (43). These aggregated states of the C-subunit also recruit C-subunits through a noncanonical mechanism and in addition create a sequestered halo of cAMP. The enhanced polymerization state of RNA that is enhanced in the CNC mutation may provide a mechanistic explanation for these “membrane-less organelles.”

## N3A motifs in the RIα and RIIβ holoenzymes

**Figure 8:**
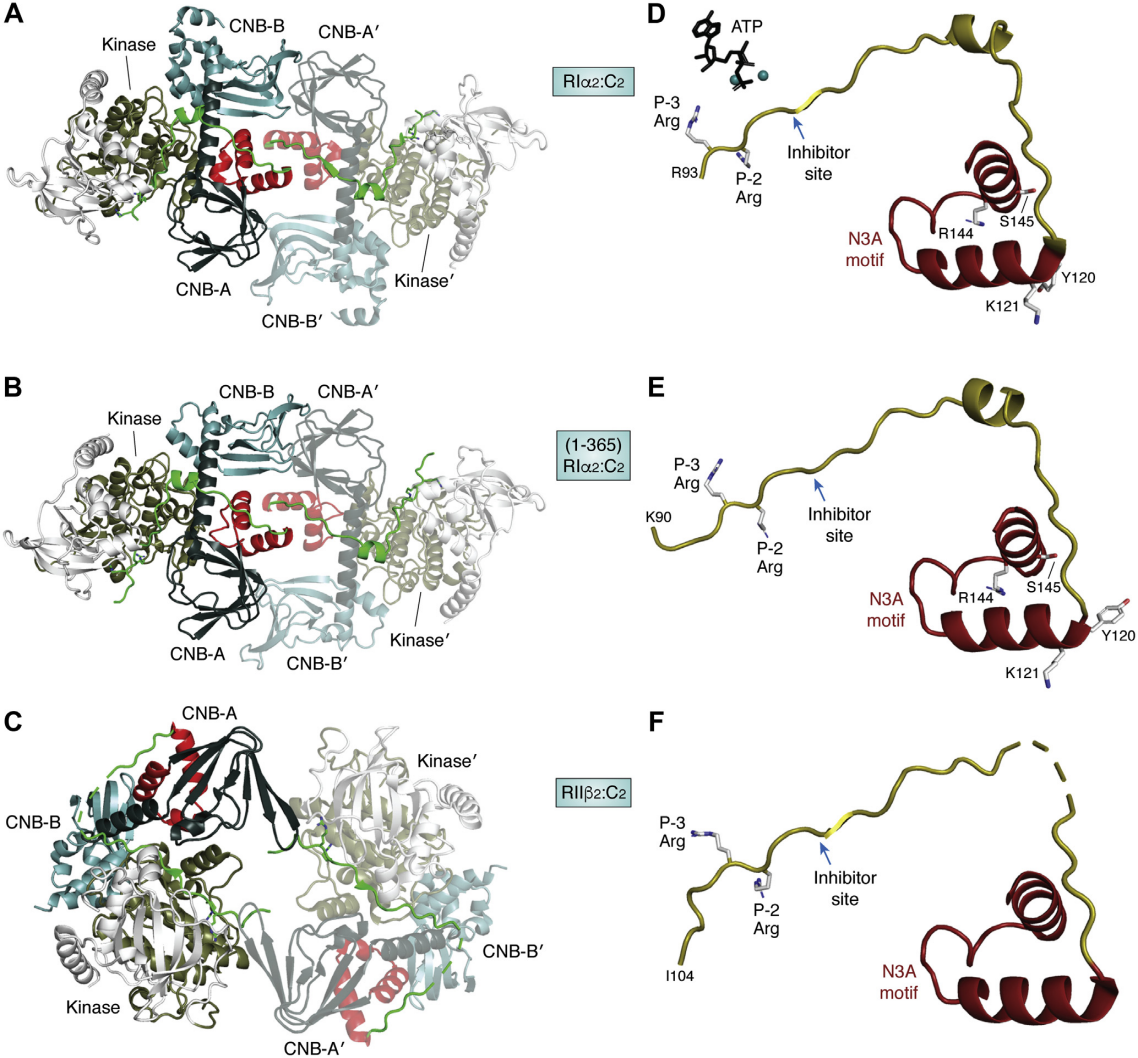
**Role of the N3A motifs in holoenzymes.** Although the N3A motifs can contribute to the organization of RIα holoenzymes, they are never seen as a dimer interface in either the free RIIα/RIIβ subunits (61, 62) or in the RIIβ holoenzymes (47, 48) where they appear to be poised to potentially interact with other proteins. *A*, an RIα holoenzyme structure shows how a highly complex and dynamic interface can be mediated by the N3A motif (44), and this organization is conserved when the RIα holoenzyme is formed with the oncogenic C-subunit that is fused to the J-domain of DNA-JB1 (45). *B*, this organization is also seen with an ACRDYS mutant of the RIα subunit where the C-terminal 14 residues are deleted rendering the holoenzyme resistant to activation by cAMP (J. Bruystens, J. Wu, J. Del Rio and S.S. Taylor, unpublished results). *C*, in the RIIβ holoenzyme the N3A motif in CNB-A interacts with its own CNB-B domain but otherwise is exposed to solvent (47). In *D* through *E* we see how the linker region from the inhibitor site to the N3A motif becomes ordered in each of these holoenzymes. *D*, in RIα ordering of the linker requires ATP (44). *E*, ACRDYS mutations that delete the C terminus also influence the requirement for ATP (J. Bruystens, J. Wu, J. Del Rio and S.S. Taylor, unpublished results). *F*, in the RIIβ holoenzyme ATP is not required for ordering of this N-linker region (44). The inhibitor site (P-site residue) is indicated by the *blue arrow*.

The organization of the tetrameric R_2_C_2_ holoenzymes allows us to appreciate the uniqueness of each isoform, and these differences are amplified when one explores the role of the N3A motif. The N3A motif is a dominant dimer interface in the RI subunits, whereas it is not a dimer interface in the RII subunits, which both have an extended conformation extended on SAXS with little direct communication between the two protomers. What about the role of the N3A motif in the holoenzymes? Several conformations of the RIα holoenzyme have been solved, and in several states the N3A dimer mediates direct cross talk between the two CNB-A domains in the two protomers (Fig. 8*A*) (44). This organization is also seen in the holoenzyme that is formed with the C-subunit that is fused to the J domain of DNAJB1, a protein that is the driver of a rare liver cancer, fibrolamellar hepatocellular cancer (45). It is surprising that this conformation is also seen in some of the ACRDYS mutants of the RIα subunit that include deletions at the C terminus (Fig. 8*B*). Our initial low-resolution structures show that ATP binding is also compromised in these holoenzyme structures, which are highly resistant to activation by cAMP (J. Bruystens, J. Wu, J. Del Rio and S.S. Taylor, unpublished results). How ATP binding is coupled to cAMP binding is clearly an essential feature of allosteric activation of the RIα holoenzyme. The N3A motif is a common feature of all R-subunits (Fig. 8*D*-*F*) but the isolated motif alone does not explain the allosteric potential of PKA signaling. The unique and isoform-specific allosteric complexity of the PKA holoenzymes is another major concept that we missed and what we have learned over the intervening years since those first PKA structures were solved and will be comprehensively addressed in collaboration with Jean Pierre Changeux in a separate paper (manuscript in preparation).

As seen in Figure 8*C*, the N3A motif in the RIIβ holoenzyme is not part of the dimer interface. Instead, it is mostly exposed to solvent where it is poised to mediate potential interactions with other proteins that contribute to unique PKA signaling islands that are nucleated at membranes by specific AKAPs.

## Cryo-electron microscopy of the RIIβ holoenzyme

**Figure 9:**
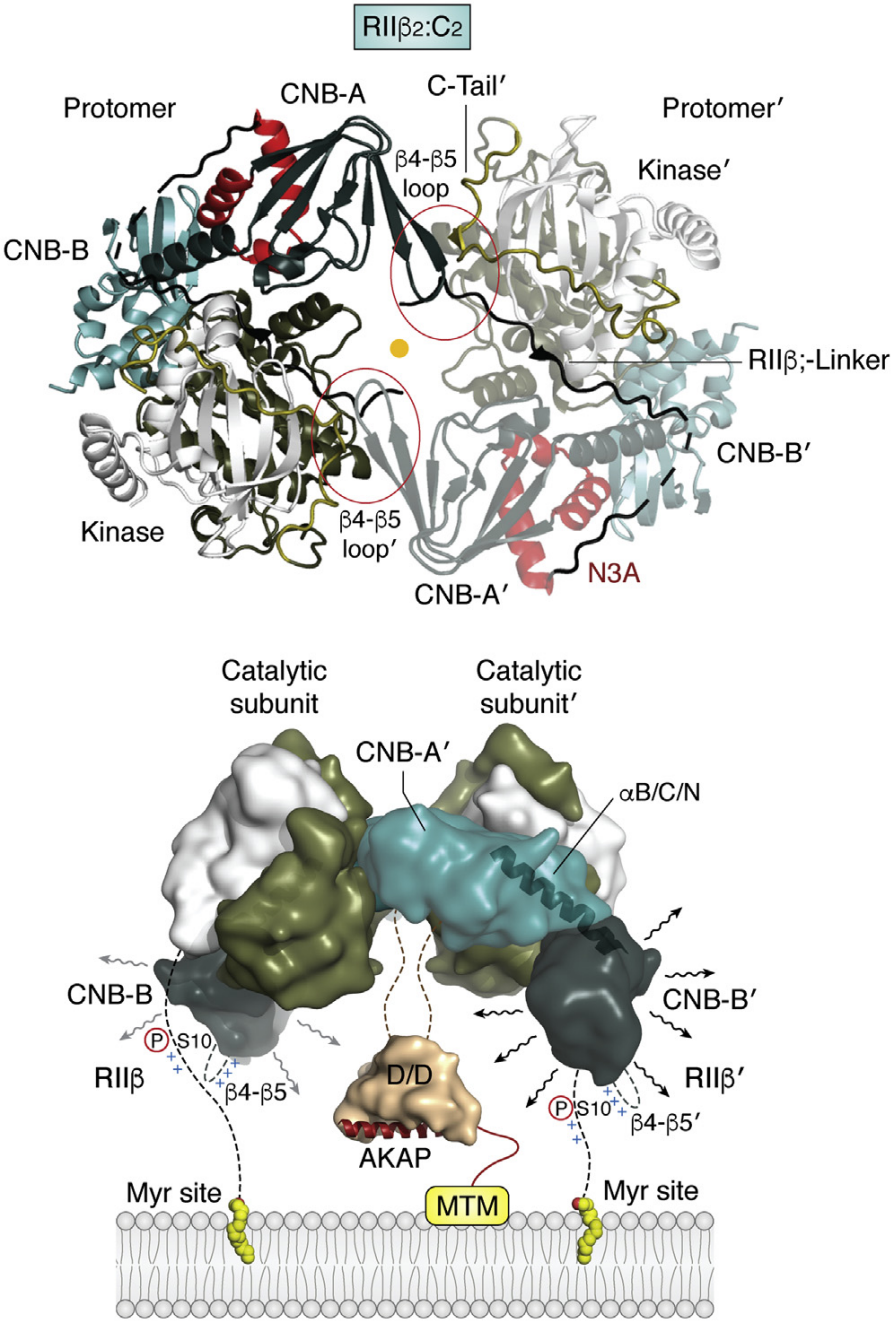
**The kinase domain is packaged in an inactive R**_**2**_**C**_**2**_**holoenzyme.** The activity of the catalytic subunit is trapped in an inactive state by cAMP-binding regulatory subunits. The full-length RIIβ holoenzyme captures the complex isoform-specific allosteric cross talk between the domains. *Top*, the cross talk between the R:C protomers is captured in a crystal lattice (47). The β4-β5 loop of one CNB-A domain (*black*) is packed against the C-terminal tail of the opposite C-subunit (*tan*) thereby assembling a complete ATP-binding pocket in the absence of nucleotide. The R_2_C_2_ complex shows an axis of symmetry (*yellow dot*) that cannot be captured in a monomeric R:C complex. *Bottom*, the complex assembly of the full-length protein, captured with cryo-EM (48), shows how the linker region that joins the N-terminal dimerization/docking domain to the cyclic nucleotide binding (CNB) domains is woven between the two protomers placing the A kinase anchoring proteins (AKAP) docking surface on the same side as the myristyl groups that are attached to the N terminus of the C-subunit. This surface is positioned to interact with membranes by utilizing a polyvalent mechanism that includes the myristyl groups at the N terminus of the C-subunits (49).

The R_2_C_2_ complex of the RIIβ holoenzyme, based on our early SAXS data (35, 46), is the most compact of the four holoenzymes, and our crystal structure of this holoenzyme first captured the exquisite allosteric cross talk between the two protomers (Fig. 9*A*) (47). It explains why the full allosteric portrait simply cannot be captured in an R:C complex but requires the full-length tetramer even if the N-terminal DD domain is not visible. This structure also shows clearly that the dimer interface is made up of the β4-β5 loop in CNB-A and the C-terminal tail of the C-subunit; the N3A motif is *not* part of this interface but instead is poised to potentially interact with another protein.

Our most recent cryo-EM structure is the first cryo-EM structure of any PKA holoenzyme (48). Although the resolution is lower, this structure nevertheless captures the organization of the full-length R_2_C_2_ holoenzyme where the linker joining the DD domain to the CNB-A domain is woven between the two R:C protomers (Fig. 9*B*). This leaves the myristyl groups at the end of each C-subunit poised to associate with membranes, and myristylation alone is sufficient to anchor the RIIβ holoenzyme to membranes, in contrast to the free C-subunit and the type I holoenzymes (49). The AKAP binding surface of the DD domain would also be close to the membrane in this model. The cryo-EM structure captures some of the complex organization and dynamics of the holoenzyme including an intrinsic asymmetry between the two RC protomers and allows us to appreciate the complexity of the cross talk between the two protomers and why it is so challenging to capture the dynamics and full quaternary features of this holoenzyme complex. Also, it shows why X-ray crystallography alone is not sufficient to capture the complex dynamics of the cross talk between the R and C subunits.

## Interdisciplinary teams and tools

Our evolving understanding of the complexity of PKA signaling highlights the fundamental importance of interdisciplinary tools. Each of us sees a protein from our own perspective—as a mechanistic driver of biology, as a drug target, as a dynamic molecular switch, as a driver of allostery, as a mediator of protein:protein interactions. To delve into each of these dimensions requires interdisciplinary teams and a large set of tools for each discipline. Although we originally thought of proteins as relatively rigid bodies, we now appreciate that they are highly dynamic molecules and that some regions are actually metastable allowing them to adopt different conformations and seek different binding partners depending on the creation of second messengers, posttranslational modifications or other transient signaling events. The sequences of these metastable regions, often referred to as “intrinsically disordered regions,” can be computationally identified (50) and are often some of the most critical mediators of biological signaling events. The crystal structure representing the highest resolution information becomes a starting point for anyone to use their own tools to explore the exciting stories that are embedded within each protein. Having these structures in the PDB allows anyone to use the information that is captured in that structure, and even today we are still learning new things about that original PKA structure.

## Conclusions and future challenges

This has been an amazing journey over 3 decades trying to elucidate the essential features of how the protein kinases have evolved to be one of the most important molecular switches in biology and then to understand how they are precisely activated in response to transient biological cues. So many diseases result when this finely tuned process is disrupted even slightly. The whole extended signaling network of the cell can be so easily reprogrammed by a single mutation in a single kinase, which is only one node in the network. And we could not have traveled this pathway so efficiently without the PDB as a resource where all data were shared. It has been and continues to be an essential tool for all of us in the signaling and structural biology communities. Although we thought that much was solved with that first structure of the PKA C-subunit, we realize now that it was only the beginning; we are still only halfway through our journey at best. Many challenges can now be met with cryo-EM, combined with crystallography and NMR, and with our powerful new computational tools. It will require this “interdisciplinary village.” And it will require us to “think outside the box” beyond the canonical mechanisms to appreciate new ways that molecules communicate with the context of cells and tissues. Ultimately our goal is to understand biology and to understand how biology is disrupted by disease. This is what we are trying to capture and understand with our structures.

## Supporting information

This article contains supporting information.

## Conflict of interest

The authors declare that they have no conflicts of interest with the contents of this article.
